# Building an enabling environment and responding to resistance to sexuality education programmes: experience from Jharkhand, India

**DOI:** 10.1186/s12978-020-01003-9

**Published:** 2020-10-30

**Authors:** Marina Plesons, Aarushi Khanna, Mohammed Ziauddin, Aparajita Gogoi, Venkatraman Chandra-Mouli

**Affiliations:** 1 Department of Sexual and Reproductive Health and Research and the Human Reproduction Programme, World Health Organization, Genève, Switzerland; 2 The David and Lucile Packard Foundation, New Delhi, India; 3 Centre for Catalyzing Change, New Delhi, India

**Keywords:** Sexuality education, Adolescents, India

## Abstract

**Background:**

Despite the substantial need for sexuality education and evidence on its effectiveness, implementing organisations continue to grapple with numerous challenges, especially related to community support and resistance. This article aims to analyse the experience of *Udaan,* a programme that has achieved remarkable success in Jharkhand, India, to answer the following questions: (1) What strategies did *Udaan* use to create a supportive environment? and (2) What processes did *Udaan* use to respond to resistance during its implementation?

**Methods:**

We reviewed programme documents and publications, synthesized key themes, identified questions of interest, and conducted interviews with key informants from the Centre for Catalyzing Change’s leadership.

**Results:**

Community support for *Udaan* was built by ensuring that the curriculum was responsive to the context, capitalizing on an enabling policy environment, institutionalizing *Udaan* through government-led implementation, prioritizing careful selection and training of teachers, emphasizing monitoring and evaluation, and engaging with community gatekeepers. *Udaan* effectively responded to resistance by organizing a formal curriculum review, orienting editors of local newspapers on the programme; responding to questions and concerns; and proactively creating positive visibility.

**Conclusion:**

The lessons from *Udaan* provide insight into approaches that can be used to design and sustain sexuality education programmes in complex settings.

## Plain English summary

Despite the substantial need for sexuality education and evidence on its effectiveness, implementing organisations continue to grapple with numerous challenges, especially related to community support and resistance. This article aims to analyse the experience of *Udaan,* a programme that has achieved remarkable success in Jharkhand, India, to answer the following questions: (1) What strategies did *Udaan* use to create a supportive environment? and (2) What processes did *Udaan* use to respond to resistance during its implementation? Community support for *Udaan* was built by ensuring that the curriculum was responsive to the context, capitalizing on an enabling policy environment, institutionalizing *Udaan* through government-led implementation, prioritizing careful selection and training of teachers, emphasizing monitoring and evaluation, and engaging with community gatekeepers. *Udaan* effectively responded to resistance by organizing a formal curriculum review, orienting editors of local newspapers on the programme; responding to questions and concerns; and proactively creating positive visibility. The lessons from *Udaan* provide insight into approaches that can be used to design and sustain sexuality education programmes in complex settings.

## Background

*Udaan*, which means “to soar in flight” in Hindi, is a school-based adolescent education programme (AEP)^1^ that has achieved remarkable success in Jharkhand, India [1]. Since 2006, the Department of School Education and Literacy, Government of Jharkhand, with technical support from a non-profit organisation called Centre for Catalyzing Change (henceforth referred to as C3), has scaled-up *Udaan* in all government secondary schools in the state. The programme has been sustained over time and extended to upper primary schools and Kasturba Gandhi Balika Vidyalaya (KGBVs)^2^ to reach a total of over 800,000 students to date. Its impact has been evaluated by independent research agencies, which have found positive effects on students’ knowledge about gender equity, sexual harassment and abuse, substance abuse and legal ages for marriage; and improvements in their self-efficacy, confidence in handling peer pressure, and communication and decision-making skills [2]. *Udaan* is one of the largest interventions of its kind in India and was recognised by national and state governments as a model programme for replication in other states [3]. Furthermore, in 2007, when India faced a wave of backlash to CSE in the media, promoted by conservative groups opposed to addressing sexuality amongst adolescents and supported by parents and community members concerned about what their children were being taught in schools, *Udaan* was able to be sustained and even subsequently expanded while similar programmes were banned in 12 other states [1].

Since the 1990s, the importance of delivering comprehensive sexuality education (CSE) to adolescents and promoting gender equality at an early age has been established and emphasized, both by research and epidemiological evidence and decades of programmatic learning [4, 5]. Furthermore, it is widely recognised that CSE is an important tool to build essential life skills (such as communication, agency, decision-making, and the ability to seek appropriate support and advice) and promote the adoption of healthy sexual practices [6]. Despite the substantial need for CSE and proven effectiveness, there are challenges that implementing organisations and policy-makers continue to grapple with, especially related to building community support for CSE and responding to resistance [7]. While this topic is frequently noted as a major barrier to CSE (and AEPs in India), there is a lack of research and detailed discussion on specific strategies that can be used to overcome it.

**Fig. 1:**
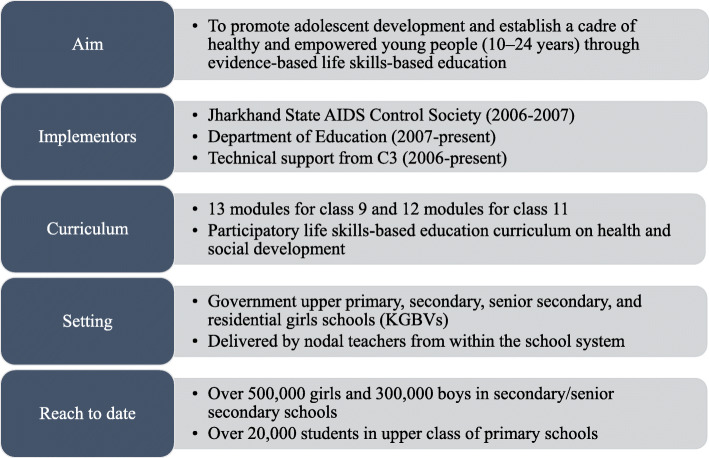
Details of the *Udaan* programme

Descriptions of *Udaan*, its theory of change, its impact among young people, and the factors that contributed to its successful scale-up have previously been documented (Fig. 1) ([1, 2]; Additional file 1). As part of an ongoing effort by the World Health Organization (WHO) to understand and document strategies for creating an enabling environment for CSE and overcoming resistance, this article aims to analyse C3’s experience in designing, implementing, and managing *Udaan* by answering the following research questions: [2, 8, 9]

1. What strategies did the Government of Jharkhand and C3 use to create a supportive environment for *Udaan* and enable it to survive in a context where many other programmes failed to do so?
2. What processes did the Government of Jharkhand and C3 use to respond to resistance that emerged during *Udaan's* implementation?

## Methods

### Data collection

A mixed approach was applied to understand the strategies adopted by the Government of Jharkhand and C3 to create an enabling environment for *Udaan* and overcome resistance. A review of programme-related literature and documentation (both published and grey literature) was undertaken to gather information and understand the Government of Jharkhand and C3’s strategies to build support and respond to resistance during its planning, implementing and scaling-up stages. The literature review identified a set of questions for further inquiry to better capture the nuances of the *Udaan* experience, with particular attention to the aspect of resistance. Key informant interviews were conducted with three individuals from C3 who were involved in the design and management of *Udaan* to respond to the questions for further inquiry.

### Data analysis

Relevant information was extracted from the programme-related literature and documentation. Themes that emerged from the literature review were supplemented with insights from the key informant interviews. Further, the engagement of C3’s executive director and its Jharkhand state programme coordinator from 2006 to 2017 as co-authors aided with the interpretation of the findings identified through the literature review and key informant interviews. To answer the two overarching research questions, the findings were organized according to four sub-questions: two of which relate to information necessary to understand *Udaan’s* context and design process (Q1 and Q2), one of which relates to proactive strategies that the Government of Jharkhand and C3 used to build support for *Udaan* (Q3), and one of which relates to the nature of the backlash that emerged and the reactive strategies that C3 used to respond to it (Q4).

1. What was the historical context that prompted *Udaan*’s development and C3’s involvement?
2. How did the Government of Jharkhand and C3 ensure that *Udaan*’s curriculum was responsive to the local context?
3. What strategies did the Government of Jharkhand and C3 use to create community support?
4. What forms of resistance did *Udaan* face during its implementation and how did the Government of Jharkhand and C3 respond?

## Results

### What was the historical context that prompted *Udaan*’s development and C3’s involvement?

**Fig. 2:**
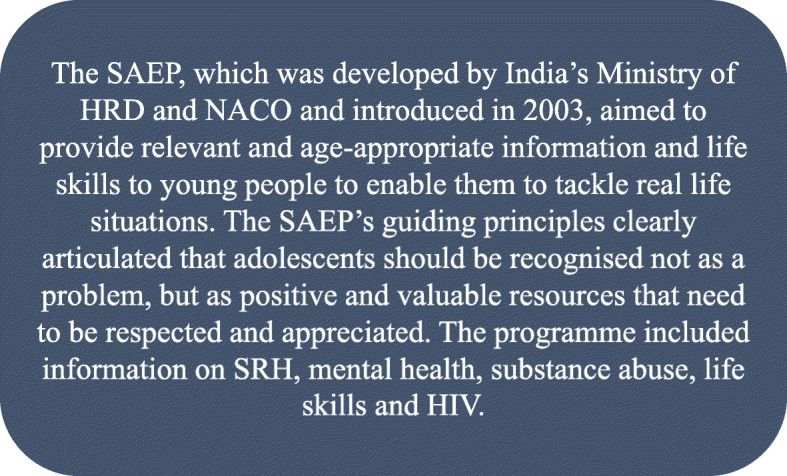
Characteristics of the SAEP

Amidst growing concerns around HIV/AIDS in the country, the Government of Jharkhand recognized the acute need for school-based sexuality education. In response, the state government designed and implemented a School AIDS Education Programme (SAEP) in 2003 (Fig. 2). The curriculum, called ‘Learning for Life’, was developed by India’s Ministry of Human Resource Development (HRD) and the National AIDS Control Organization (NACO), with support from the National Council on Education Research and Training (NCERT), UNICEF, and UNESCO, and was designed to focus on HIV prevention and to address adolescents’ vulnerabilities related to risky sexual behaviour. The SAEP was implemented state-wide over a three -year period with students from classes 9 and 11, led by the Jharkhand State AIDS Control Society (JSACS) and the NACO. In 2006, the state government recognized that the SAEP was not addressing the comprehensive needs of adolescents, including a range of issues beyond HIV such as poor access to and use of sexual and reproductive health (SRH) services and high rates of early marriage. In response, the Government of Jharkhand created a state-specific youth health policy, one of the few of its kind in the country, and decided to expand the SAEP to a broader programme on developmental wellbeing in 2006, called *Udaan*.

Understanding the useful role of NGOs in providing technical assistance, JSACS sought a credible partner with experience of working in Jharkhand to support the development, implementation and management of *Udaan*. They identified C3, which was then leading the implementation of the Better Life Program (BLP) in 11 states in India, including Jharkhand. After a thorough review and assessment, the Project Director of JSACS invited C3 to provide technical oversight for improving and modifying *Udaan's* curriculum to comprehensively respond to adolescents' sexual and reproductive health needs, build state capacity, and mainstream the initiative through the Department of Education (DoE). Since 2006, C3 has served as the technical partner to the Government of Jharkhand on the implementation and scale-up of *Udaan*.

### How did the Government of Jharkhand and C3 ensure that *Udaan*’s curriculum was responsive to the local context?

Prior to the development of *Udaan*’s curriculum, C3’s undertook an evaluation of the SAEP’s ‘Learning for Life’ curriculum. Concurrently, C3 conducted a needs assessment using individual interviews and focus group discussions with master trainers (MTs), nodal teachers (NTs), principals, and secondary school students at nine schools in Ranchi and neighbouring districts, as well as with parents, relevant NGOs, media professionals, and government officials.

This process resulted in C3’s recommendation to the Government of Jharkhand to adapt and revise the ‘Learning for Life’ curriculum. Specifically, the needs assessment results indicated a need to increase the duration of MT and NT trainings; revise the training methodology to be more participatory; conduct refresher trainings; tailor training manuals and curricula to be age- and context-specific; expand the curricula to include issues related to life skills, adolescent health, nutrition, and common diseases; increase the curricula to 20 h; emphasize principals’ involvement in programme implementation; and establish a reporting system with feedback mechanisms. Although the evaluation was time and labor intensive, this process was both crucial and fruitful to ensuring that the programme’s content and implementation modalities were suited to the context.

Next, the Government of Jharkhand and C3 created a Core Committee in 2006 with representatives from the JSACS, the DoE, the academic council, and C3, as well as MTs, NTs, and secondary school students. A participative process with this group of stakeholders was undertaken to incorporate the findings of the needs assessment to create a revised, comprehensive, age-appropriate 13-module curriculum for class 9 and a 12-module curriculum for class 11. The curricula included content on a range of issues including goal setting and values; growing up; communal harmony; friendship and peer pressure; gender and sexual harassment; substance abuse; marriage and parenthood; early marriage and its consequences; and SRH including contraception and reproductive tract infections (RTIs)/sexually transmitted infections (STI), including HIV/AIDS. Throughout each module, the curricula incorporated exercises to develop life skills – an important element that distinguished *Udaan* from other AEPs in the country at the time. Likewise, participatory, non-judgmental teaching methodologies were emphasized through the inclusion of games, stories, case studies, and quizzes. To support teachers to use these participatory methodologies, C3 led the development of a 20-h training package for class 9 teachers and an 18-h training package for class 11 teachers, as well as training/reference manual.

To ensure that the curriculum development process incorporated the perspectives of diverse stakeholders, the Government of Jharkhand and C3 hosted a three-day materials development workshop in June 2006. The attendees reviewed the curriculum and supplementary materials. They recommended minor changes to the content and language, which were accommodated by C3. The revised curriculum and supplementary materials were then field-tested in three schools in Ranchi district, reviewed by stakeholders, and approved by the JSACS and DoE. The curriculum has also been updated from time to time in response to findings of ongoing monitoring, periodic evaluations, and feedback from the Core Committee, and in response to revisions to national and state policies on adolescent education. The curriculum for classes 6, 7, and 8 were last updated in 2019.

### What strategies did the Government of Jharkhand and C3 use to create community support?

**Fig. 3:**
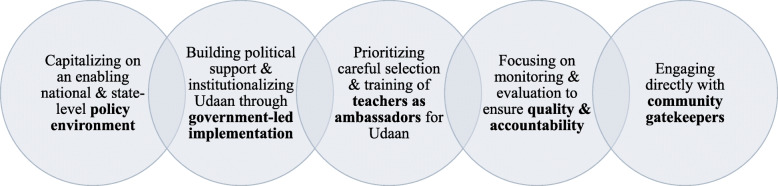
Key strategies used by C3 to create community support for *Udaan*

The Government of Jharkhand and C3 created community support for *Udaan* by capitalizing on an enabling policy environment, institutionalizing the programme through government-led implementation, ensuring teacher capacity, prioritizing quality improvement, and engaging directly with community gatekeepers (Fig. 3).

#### Capitalizing on an enabling national and state-level policy environment

The creation of community support for *Udaan* was underpinned by its enabling national and state-level policy environment, as well as the Government of Jharkhand and C3’s strategies to leverage these mandates to support the programme’s implementation and scale-up. As early as 1986, the National Policy on Education underscored the importance of educating young people about family planning and responsible parenthood. The National Population Policy (2000), the National AIDS Prevention and Control Policy (2002), and the National Health Policy (2002) reiterated a focus on young people’s sexual behaviour, reproductive health and rights, and gender equity [10–12]. Most recently, the National Youth Policy (2014) explicitly articulated the need for establishing adolescent clinics and providing free, state-sponsored counseling services for young people [13]. At the state level, Jharkhand was one of only a few states to create a state-specific Youth Policy (2007), which supported the empowerment of young people, promotion of the adolescents’ reproductive health and rights, introduction of school-based Family Life Education, and reduction of adolescents’ vulnerabilities [14].

Furthermore, the Indian government issued a national directive and operational guidelines for school-based SRH education programmes in all secondary and senior secondary schools in 2004, in response to concerns about HIV/AIDS [15]. As a result, the implementation and scale-up of *Udaan* benefitted from a predetermined mandate.

#### Building political support and institutionalizing *Udaan* through government-led implementation

C3 understood that building and sustaining political support through government-led implementation was a key strategy to promote community support for *Udaan*’s implementation and scale-up. The programme was launched by the chief minister of the state, demonstrating from the beginning that the programme was completely owned by the government. Further, the Core Committee (described above), which was set up to advise the design of *Udaan*, included representation from government, as well as a number of other stakeholders. Additionally, C3 conducted consistent state-level advocacy with state functionaries to build and sustain political support for *Udaan*. At the district level, C3 held regular advocacy meetings with district education officers (DEOs) and related coordinators. On an annual basis, interaction and orientation were also conducted with school principals, community leaders, parents, and media professionals on the programme and its curriculum.

*Udaan* was originally implemented in government schools by JSACS; however, this arrangement encountered challenges related to implementing a school-based intervention through a health department. Therefore, to stimulate ownership of the programme and deliver the programme cohesively within the academic timetable, the Government of Jharkhand transferred responsibility of the programme from JSACS to Jharkhand’s DoE in April 2007.

Additionally, because *Udaan* was implemented within government schools by teachers that parents knew and trusted, the programme was able to attain a degree of credibility and perceived value simply through its association with the school system. Furthermore, the technical support from C3 and the financial support to C3 from the David and Lucile Packard Foundation enabled long-term and consistent institutional support for *Udaan* to take root and attain scale.

#### Prioritizing careful selection and training of teachers as ambassadors for *Udaan*

Recognizing that teachers are the face of AEPs from communities’ perspectives, the Government of Jharkhand and C3 placed emphasis on ensuring that the teachers were exemplary ambassadors for the programme. In 2006, a cascade training approach was used for the implementation of *Udaan*. First, a cadre of school principals from government secondary and senior secondary schools across the state were selected and oriented on the programme. The DoE and JSACS, with support from principals, developed selection criteria for MTs, and teachers were nominated. Those who were nominated participated in five-day, 50-h residential training workshops on technical issues in groups of 18–20, followed by several rounds of practice sessions to develop training skills.

Meanwhile, each principal selected two NTs on the basis of their credentials and interest in adolescent development, who were subsequently trained by the MTs and C3’s technical experts through four-day residential training workshops in groups of 30–35. Trainings were held during summer breaks, and the state DoE agreed to provide compensatory time-off for attending training during vacations. In October 2009, *Udaan*’s curriculum was also introduced in selected Bachelor of Education (B.Ed.) colleges for pre-service teachers’ training.

#### Engaging with community gatekeepers

In addition to its emphasis on teachers, the Government of Jharkhand and C3 engaged directly with gatekeepers at many levels to build community support. To gain political buy-in from government officials, DEOs were formally designated as district nodal officers. As a result, discussion on *Udaan* was integrated into the agenda of the DEOs’ monthly meetings. One-day district-level orientation meetings were initiated in September 2006 to sensitize district-level stakeholders, including DEOs in all 22 districts and 444 principals. Likewise, a cadre of *Udaan Mitras* (DEO clerical staff) were designated as point persons to assist with *Udaan*’s management and coordination, and were then oriented alongside the DEOs, MTs, and regional deputy directors of education.

Transparency of *Udaan* and engagement of parents, community members, and community leaders was ensured through formation of *Udaan* clubs and organization of *Udaan* mahotsavs (festivals/fairs). *Udaan* clubs leveraged pre-existing red ribbon clubs (originally created by NACO to build awareness of HIV) as a platform and were used to ensure that the *Udaan* curriculum was shared beyond class 9 and 11 students. *Udaan* mahotsavs, held in five administrative regions in 2012, for example, brought together students, NTs, principals, and district-level officials. They recognized and awarded best-performing schools, and helped to create visibility and understanding of the programme.

#### Focusing on monitoring and evaluation to ensure quality and accountability

Community resistance to CSE can be conceptualized like wildfire: often all it takes to ignite resistance is a small spark. As a result, the Government of Jharkhand and C3 understood that continuous quality improvement was critical to sustaining *Udaan*’s community support. It thus prioritized regular and timely collection of data on quality and fidelity to track the programme’s implementation and impact. An MIS system was implemented to support state, district, and school-level monitoring, and C3 piloted a low-cost technology to collect monthly data on *Udaan* sessions using an Interactive Voice Response System (IVRS).^3^ Monthly monitoring was conducted at district-level principals’ meetings and at state-level DEOs’ meetings. At the end of the first phase of implementation, C3 helped the DoE to conduct a quasi-experimental post-intervention evaluation to assess *Udaan*’s implementation. Two post-intervention impact evaluations were conducted in October 2008 and November 2009 to assess *Udaan*’s impact, and an evaluation of the implementation and integration of the MIS was conducted in July 2010 [1].

The Government of Jharkhand and C3 also understood that data can be a powerful advocacy tool. Transparency and dissemination of the results of the first evaluation was ensured through a mid-term sharing meeting, which was held in 2007 and was attended by over 200 participants, including DEOs, a regional deputy director, deputy directors, NGO representatives, media personnel, principals, MTs, NTs, and students. In 2008, NACO coordinated a national review meeting of AEPs in New Delhi, where C3 and the Government of Jharkhand were requested to present the *Udaan* model. Based on this presentation, NACO identified the *Udaan* model as a good practice and recommended that it be implemented in all states. In 2016, the Ministry of Health and Family Welfare selected *Udaan* as a “Good and Replication Practice and Innovation in India”. Public recognition and acclamation such as these helped to garner support for *Udaan* and similar programmes [16].

Furthermore, the Government of Jharkhand and C3 used evaluation results and feedback from stakeholders, especially students, to inform and shape programmatic decisions. For instance, a midcourse evaluation in October 2010 to assess the impact of *Udaan* on students’ knowledge, attitudes, perceptions, and intentions found that students in class 11 had lower interest and behavioural changes than class 9 students. Based on this finding, *Udaan* was expanded to reach students in upper primary classes (classes 6, 7, and 8). Allowing students’ voices and stakeholders’ inputs to hold weight in the DoE and C3’s decisions helped to continually refine the programme and create a sense of ownership of *Udaan* among its stakeholders.

### What forms of resistance did *Udaan* experience during its implementation and how did the Government of Jharkhand and C3 respond?

Despite C3 and the Government of Jharkhand DoE’s concerted efforts to create an enabling environment and ensure institutionalization of *Udaan*, the programme encountered resistance.

In 2007, backlash to CSE increased across the country. Conservative groups claimed that the CSE curriculum promoted by India’s Ministry of HRD was against traditional Indian values and would encourage young people to engage in promiscuous and irresponsible behaviour. These claims were bolstered by parents and community members concerned about what their children were being taught in schools. In response, 12 states banned all AEP and CSE programmes. Although the Government of Jharkhand did not ban CSE, it also experienced backlash to *Udaan* as part of this controversy. Media in the state fuelled misgivings among parents and community members about the delivery of the programme in government schools and instigated communities’ fears that *Udaan* would encourage sexual experimentation among adolescents.

JSACS and DoE, in collaboration with C3, adopted a series of strategic measures to promptly respond to the backlash. First, an immediate review of the AEP and its curricula was undertaken. Secondly, both departments restricted mid-management officials’ interactions on *Udaan* with media; further, C3 was directed to keep a low profile, to ensure that the programme was communicated and perceived as being government-led. C3 provided the DoE with data in support of the need for and impact of *Udaan*, while the DoE led the response and reaction process. As a primary measure to counter misinformation, joint press statements and clarifications were issued by the Minister of HRD and the Secretary and Project Director of JSACS. This action conveyed a strong message about the government’s confidence in and commitment to the programme and its curricula. JSACS and the Ministry of HRD also prepared themselves for possible questioning and comments on *Udaan* from political parties at the State Assembly’s monsoon session, using data and evidence available about the need for and impact of the programme.

To directly address media concerns and bring about change in public perception of AEPs, the Project Director of JSACS oriented editors of all local newspapers on the importance of *Udaan* and responded to questions and concerns about its content. JSACS also provided copies of programme materials to the editors, and invited them to district-level trainings and other events. As a result, the journalists published positive articles on the importance of *Udaan,* and on the fact that the curricula was designed to respond to the specific needs of adolescents in Jharkhand. Such journalists have continued to play an important role in holding the Government of Jharkhand and C3 accountable for the programme’s fidelity to its intended design.

To communicate transparency of *Udaan*, the previously-described state-level mid-term sharing meeting was organised, and the DoE and JSACS used the opportunity to share the merits of *Udaan* and challenges associated with implementation and scale-up. Meanwhile, no concerns were raised by participants about the content of the programme. The meeting effectively changed the perspective of local newspapers, which had reported negatively on *Udaan*; after the meeting, it published positive stories on the programme. The ownership of the programme by the Government of Jharkhand DoE and the buy-in of multiple stakeholders created a buffer between *Udaan* and the wave of national backlash to AEPs and helped to re-establish a supportive environment in the state.

Learning from this experience of media-related backlash, the Government of Jharkhand and C3 took proactive actions to create greater visibility and increase positive public support for *Udaan* to avoid future backlash or resistance. C3 facilitated regular sensitization workshops at state and district levels to gain buy-in from a range of relevant stakeholders including community leaders, parents, teachers and media personnel. Immediately after such workshops, several journalists published positively about *Udaan*. Building upon this positive momentum, the Government of Jharkhand and C3 also leveraged important occasions, such as World AIDS Day and Jharkhand Foundation Day, to advocate for *Udaan*. Additionally, they used *Udaan* mahotsavs in each administrative region to allow stakeholders to share experiences and stories about their involvement with *Udaan*.

Thus, negative reactions from the media were handled successfully as a result of the close collaboration and trust between the Government of Jharkhand and C3, along with their commitment, involvement, and sense of ownership towards *Udaan*. While the backlash that occurred in 2007 was not anticipated, the organisations’ immediate response and their initiative to put measures in place to avoid future resistance have proven effective and negative reactions of this magnitude have not re-surfaced. These efforts were instrumental in ensuring that while AEPs were banned in so many states, delivery of *Udaan* continued in Jharkhand.

## Discussion

This analysis identified key strategies that the Government of Jharkhand and C3 employed to create a supportive environment for the effective implementation and scale-up of *Udaan*. First, the Government of Jharkhand and C3 ensured that *Udaan*’s curriculum was responsive to the local context, capitalized on an enabling national and state-level policy environment, institutionalized *Udaan* through government-led implementation, prioritized careful selection and training of teachers as ambassadors for the programme, focused on monitoring and evaluation to ensure quality and accountability, and engaged with community gatekeepers. Despite these efforts, *Udaan* did face backlash during the course of its implementation. However, the Government of Jharkhand and C3 undertook activities to respond to this resistance, including organizing a formal review of the curriculum by the DoE, press statements by high-level officials, and a state-level mid-term sharing meeting; orienting editors of all local newspapers on the significance and need for *Udaan* and responding to questions and concerns about its content; and implementing proactive actions to create greater visibility and increase positive public support for *Udaan* to avoid future backlash or resistance. As a result, *Udaan* achieved what many programmes in India and around the world have failed to do: it successfully scaled-up and sustained the delivery of age-appropriate, contextually-relevant, and government-run sexuality education.

The findings of this analysis on successful strategies for building community support for CSE and overcoming resistance are similar to findings from other contexts. For example, the usefulness of the public-private partnership between the Government of Jharkhand and C3 – whereby the programme can benefit from governments’ inherent credibility and authority and its schools as an existing delivery platform on the one hand, and NGOs’ ability to nimbly navigate contentious waters while offering enhanced technical support on the other – match similar experiences from Senegal and Mexico [17, 18]. Similarly, the choice to label *Udaan* as an AEP instead of CSE echoes choices made by many programmes in other contexts, such as use of “family life education” or “reproductive health education” in Senegal, “family life and HIV education” in Nigeria, and “life skills based education” in Pakistan [8, 9, 17, 19, 20]. One factor that differed in the case of *Udaan* as compared to findings from other contexts is political support: *Udaan* benefited from a state government (and specifically the JSACS and the DoE) which recognized the SRH needs of its adolescents, believed that CSE could help to address these needs, was fully committed to owning the programme in partnership with a credible and competent NGO, and was not afraid to stand up to opposition when backlash arose. This enabled *Udaan* to move forward effectively, despite the expected ups and downs of programme implementation, while programmes in contexts where political support was lacking struggled to do so. Likewise, another strategy used by other programmes – namely addressing backlash or resistance by engaging with religious leaders – was not as pronounced with the case of *Udaan,* given that the resistance that emerged was not explicitly grounded in religious ideology or driven by religious leaders [1, 3, 17, 20]. Programmes that have done this have used strategies such as including references to religious texts within programmatic materials, or compromising on content about especially contentious issues [8, 9, 17, 20].

This analysis is limited to the experiences of one NGO operating in collaboration with government in one state in India; as such, we recognize that the lessons learned from the Government of Jharkhand and C3’s efforts are grounded in their contextual environment. However, given the lack of published evidence on strategies that can be used to anticipate and overcome resistance to CSE, this analysis represents an important contribution to the literature. Additionally, the analysis was limited to the scope of information available in *Udaan*’s programmatic reports and from the insights of key C3 staff. While the analysis could have been enriched with the perspectives of other stakeholders, C3’s extensive experience in Jharkhand and its sustained involvement in *Udaan* support the centrality of their inputs.

The implications of this analysis point to the need for more dialogue on and documentation of proactive and reactive strategies that governments and organisations globally are adopting to build support for CSE and to anticipate and overcome resistance to it. Resistance to CSE is likely to be omnipresent, but the experience of *Udaan* reiterates that there are innovative strategies that can and are being used to handle opposition, both at the programme design stage and when faced with situations of backlash. The experience of *Udaan* points to a few key recommendations: contextualize and secure formal approval by relevant authorities of the curriculum content; build strategic partnerships with relevant authorities and local newspapers; invest time in community engagement; and ensure strong monitoring and evaluation so that the benefits of the programme can be shared with relevant authorities, the media, and communities. These approaches can be modified to fit the contexts of programmes around the world.

## Conclusion

Discussing adolescent SRH in the context of India comes with a range of challenges, including cultural conservatism regarding adolescent sexuality, misconceptions about CSE content, and lack of political will to meet adolescents’ SRH needs. The experience of *Udaan* points to a clear set of strategies that can be adapted and used to design and sustain CSE programmes in complex settings. Programmes in India and elsewhere can contribute to these learnings by documenting CSE implementation, the challenges it faces, and strategies used to address these challenges, in order to create a body of evidence that can be leveraged to successfully deliver CSE to adolescents.

## Supplementary information

**Additional file 1.** C3’s Theory of Change for Adolescent Programming.

## Data Availability

The datasets used and/or analyzed during the current study are available from the corresponding author on reasonable request.

## Abbreviations

AEP: Adolescent education programme
ASRH: Adolescent sexual and reproductive health
B.Ed.: Bachelor of Education
BLP: Better Life Program
C3: Centre for Catalyzing Change
CSE: Comprehensive sexuality education
DEO: District education officer
DoE: Department of Education
IVRS: Interactive Voice Response System
JSACS: Jharkhand State AIDS Control Society
MT: Master trainer
NACO: National AIDS Control Organization
NCERT: National Council on Education Research and Training
NT: Nodal teacher
RTI: Reproductive tract infection
SAEP: School AIDS Education Programme
SRH: Sexual and reproductive health
STI: Sexually transmitted infection
WHO: World Health Organization

## Footnotes

1 In India, the term ‘adolescent education programme (AEP)’ is used to refer to school-based sexuality education or life skills-based education programmes.

2 KGBVs are residential schools that provide education for girls belonging to scheduled castes, tribes, minority communities, and families below the poverty line.

3 IVRS - To facilitate effective monitoring of the *Udaan* in school, C3 developed a phone-based IVRS to gather implementation data from schools. IVRS was formally approved and rolled out by the State Department of Education. With the support of its technical partner, C3 finalized the IVRS indicators and subsequently oriented nodal teachers on how to navigate and input information into the IVRS. Calls were made to nodal teachers to invite them to use the tool. Prior to the scheduled calls, SMS notifications were flashed to the nodal teachers, in order to ensure participation as well as to ensure access to such information at the time of the call.
