# Supplementary material for: Building an enabling environment and responding to resistance to sexuality education programmes: experience from Jharkhand, India
Source: Reprod Health. 2020 Oct 30;17:168. doi: 10.1186/s12978-020-01003-9 (PMC7597059; doi:10.1186/s12978-020-01003-9)
Supplement: Supplementary file 1 — Additional file 1. C3’s Theory of Change for Adolescent Programming. [file 12978_2020_1003_MOESM1_ESM.docx]

**C3’S THEORY OF CHANGE FOR ADOLESCENT PROGRAMMING**

***Goal / Impact***

**Create an empowering ecosystem and positively impact the lives of disadvantaged adolescents in India through a comprehensive approach that impacts and improves outcomes by leveraging available scalable platforms to ensure they receive all the relevant support and services they need**

**Delayed age of first pregnancy**

**Outcomes**

**Delayed age of marriage**

**Completion of secondary education**

**Improved agency**

**Outputs**

**Improved capabilities of teachers and stakeholders for better implementation**

**System strengthened to promote issues that positively impact adolescents**

**Policy decisions influenced to improve adolescent-focused policies**

**Intermediate Outcomes**

**Adolescents have a better understanding of gender and relationships and knowledge of SRH**

**Teachers are trained to empower adolescents on informed decision-making**

**Adolescents have the confidence to access SRH services, including contraception**

**Education system is strengthened to deliver adolescent education content**

**Activities**

**Develop state-specific curricula and build the capacity of stakeholders for implementation of Udaan across the state**

**Generate evidence and learnings to strengthen programme design and delivery**

**Empower teachers with knowledge and skills to drive collective action and community transformation**

**Support and engage with the government to facilitate convergence and improve adolescent-focused policies**

**Strengthen the education system to make it more responsive towards adolescent issues**
